# Optimization Scheme for Modulation of Data Transmission Module in Endoscopic Capsule

**DOI:** 10.3390/s25154738

**Published:** 2025-07-31

**Authors:** Meiyuan Miao, Chen Ye, Zhiping Xu, Laiding Zhao, Jiafeng Yao

**Affiliations:** 1 School of Communication and Information Engineering, Nanjing University of Posts and Telecommunications, Nanjing 210023, China; meiyuanmiao@njupt.edu.cn (M.M.); zhaold@njupt.edu.cn (L.Z.); 2 School of Ocean Information Engineering, Jimei University, Xiamen 361021, China; xzpxmu@gmail.com; 3 College of Physics & Optoelectronic Engineering, Jinan University, Guangzhou 510632, China; jfyao@jnu.edu.cn

**Keywords:** endoscopic capsule, modulation scheme, WBANs, in-body

## Abstract

The endoscopic capsule is a miniaturized device used for medical diagnosis, which is less invasive compared to traditional gastrointestinal endoscopy and can reduce patient discomfort. However, it faces challenges in communication transmission, such as high power consumption, serious signal interference, and low data transmission rate. To address these issues, this paper proposes an optimized modulation scheme that is low-cost, low-power, and robust in harsh environments, aiming to improve its transmission rate. The scheme is analyzed in terms of the in-body channel. The analysis and discussion for the scheme in wireless body area networks (WBANs) are divided into three aspects: bit error rate (BER) performance, energy efficiency (EE), and spectrum efficiency (SE), and complexity. These correspond to the following issues: transmission rate, communication quality, and low power consumption. The results demonstrate that the optimized scheme is more suitable for improving the communication performance of endoscopic capsules.

## 1. Introduction

With the development of healthcare, people are no longer limited to traditional detection methods. For example, some tests that require patients’ internal examination are no longer confined to wired methods but focus more on user experience. A typical medical application is gastrointestinal endoscopy, where a patient swallows and excretes an endoscopic capsule that sends signals for detecting its internal conditions [1,2,3]. Gastrointestinal endoscopy is mainly used to help diagnose diseases such as gastritis, gastric ulcers, gastroesophageal reflux disease, intestinal inflammation, and digestive tract tumors. Through gastrointestinal endoscopy, doctors can directly observe the condition of the stomach, esophagus, small intestine, and large intestine, identifying lesions such as ulcers, tumors, and polyps for precise localization. This requires an investigation of the signal transmission quality in body area network environments [4,5,6]. An important aspect of smart healthcare is the development of communication technology for internal body channels, such as endoscopic capsules. The usage of an endoscopic capsule involves ingesting a capsule that contains communication functionality, allowing it to transmit real-time data while traveling through the gastrointestinal tract. The capsule can capture gastrointestinal images and send them to external devices for analysis and diagnosis, offering a less invasive alternative to traditional approaches like gastroscopy or colonoscopy [7]. A key challenge in this field is ensuring the communication system can effectively adapt to a patient’s internal body environment, including dealing with interference and maintaining a stable connection [8]. This is a critical area of focus in advanced smart healthcare technologies.

Wireless body area networks (WBANs) are emerging technologies that play a critical role in various fields, particularly in healthcare and medical monitoring applications. WBANs enable continuous monitoring of physiological parameters through wearable devices, providing real-time health data to clinicians for better decision-making and timely interventions [9]. These networks are composed of small, low-power devices embedded in or worn on the body, which communicate wirelessly with external devices such as smartphones or health monitoring systems. The IEEE 802.15.6 standard has been specifically designed to address the unique challenges of WBANs, including energy efficiency, low latency, and reliability in healthcare settings [3]. Recent research has focused on improving the performance of WBANs by optimizing medium access control (MAC) protocols and Physical (PHY) layer parameters. For instance, a modified IEEE 802.15.6 MAC scheme has been proposed to enhance performance in e-health applications by adjusting superframe structures and access mechanisms to better meet healthcare demands [10]. Considering a practical scenario, the channel model 2 (CM2) in the WBAN standard is a variable on-body channel model described in the IEEE. Additionally, a study by Siddik et al. investigates the effects of MAC and PHY layer parameters on the performance of IEEE 802.15.6 CSMA/CA, using a Markov chain-based model to assess network performance under various scenarios [11]. The integration of WBANs with advanced modulation schemes and energy-efficient technologies holds significant potential for improving healthcare delivery through continuous, real-time health monitoring. However, overcoming the challenges of signal interference, energy consumption, and scalability remains a critical area of research. Future developments in this field should focus on optimizing communication protocols, enhancing energy efficiency, and exploring more robust integration strategies with existing healthcare infrastructures [12,13]. Given the capsule’s compact size and the realities of patient use, what is needed is a low-cost, low-power modulation that delivers reliable communication without bulky or power-hungry filters and equalizers.

Differential chaos shift keying (DCSK) is a promising modulation technique known for its low cross-correlation, low power consumption, exceptional autocorrelation, and broadband spectral characteristics. Therefore, it can have strong robustness against non-stationary environments such as multipath interference without the need for filtering and equalization [14]. However, these advantages come at the cost of reduced system spectral efficiency (SE). To enhance the transmission rate, the implementation of the M-ary DCSK (M-DCSK) method becomes necessary. Several M-DCSK systems have been developed to achieve high data rates by combining index modulation (IM) with traditional binary DCSK [15,16,17,18,19]. Numerous methods are using the coded-shift DCSK (CS-DCSK) based on Walsh codes, introduced in [20]. Subsequent developments have led to the generalized CS-DCSK, high-data-rate CS-DCSK, and CS-DCSK with code index modulation, targeting high-speed systems, as presented in [21,22,23], respectively. These schemes offer a notable improvement in spectral efficiency by enabling the transmission of multiple bits per symbol period. Additionally, a MIMO M-ary DCSK scheme was proposed in [24]. Despite these advantages, the complexity of these schemes remains relatively high. Additionally, several schemes based on M-ary phase shift keying (MPSK) and M-ary quadrature amplitude modulation (MQAM) for DCSK have been proposed [25], with a focus on optimal constellation design. These schemes make use of a reference chaotic signal in the information-carrying part [26], which is modulated by MPSK and MQAM using M-ary symbols. Furthermore, an M-DCSK system employing an MPSK constellation [27], based on orthogonal chaotic frequency shift keying (QCSK) [28], has been proposed, demonstrating improved bit error rate (BER) performance.

In recent years, WBANs have become a crucial part of modern healthcare systems, particularly for applications such as health monitoring, wearable sensors, and medical implants. These systems typically need effective communication methods to transmit critical biomedical data. Among various modulation techniques, DCSK has emerged as a promising approach due to its robustness against noise, interference, and fading, which are common in the in-body communication channels. Ref. [29] demonstrated that chaotic secure communication systems, such as those using DCCK, offer robust security and low power consumption, making them ideal for WBAN applications. Furthermore, Li and Zhang provided an in-depth performance analysis of DCCK in WBANs, showing its potential in achieving high reliability while maintaining low energy consumption, even in complex wireless environments [30]. In addition to performance improvements, the security of transmitted data in WBANs is crucial due to the sensitive nature of health-related information. Ref. [31] proposed a secure communication framework based on DCCK modulation, emphasizing its ability to safeguard data transmission from potential eavesdropping. Their work highlights the key advantage of chaotic modulation in WBANs, offering both privacy and communication efficiency. Furthermore, Ref. [32] explored the design and performance of DCCK in WBANs, showing that it effectively reduces bit error rates and enhances the stability of data transmission in noisy environments.

Interbody (In-body) communication, where signals travel through or around the human body, presents several unique challenges such as high attenuation, multipath fading, and interference from the body itself. DCSK, which utilizes the chaotic dynamics of a signal for modulation, has demonstrated its potential to handle these challenges effectively. The chaotic nature of the signal offers high resilience to interference and provides enhanced security features, which are vital for medical and health-related applications where data integrity and privacy are crucial. Previous studies have shown that DCSK can significantly improve performance in highly dynamic and noisy environments, such as those found in in-body channels. Ref. [33] explored DCSK for body area networks (BANs) and demonstrated that this modulation scheme effectively mitigates the effects of fading and interference in in-body communication environments, ensuring reliable data transmission under adverse conditions. Furthermore, the challenges posed by highly dynamic channels are discussed, and a DCSK-based solution that performs well in fluctuating channel conditions is proposed, enhancing the communication quality and robustness [34].

In summary, DCSK offers several advantages for in-body communication systems, including resilience to channel impairments, low power consumption, and enhanced security features. These benefits make it a highly suitable candidate for the future of body-centric medical and healthcare networks.

The contributions of this paper are shown as follows:

(1) This paper first introduces the model of the in-body channel environment, as well as the channel environment model of WBANs, including the establishment of relevant models and the setting of parameters. Based on the characteristics of the in-body channel environment and the demand for transmission efficiency, a Multi-level Index Position M-ary Differential Chaos Shift Keying (MIP-MDCSK) scheme is designed. This scheme improves the transmission rate by combining index position with MDCSK, where part of the transmission bits are realized by index position and the other part is implemented by the MDCSK modulation itself. The principle of its implementation is discussed in detail.

(2) The energy efficiency (EE), spectral efficiency (SE), and complexity of the proposed scheme are derived and calculated. A comparison with other related schemes shows that the proposed approach achieves a significant improvement in both energy efficiency and spectral efficiency, albeit with a slight increase in complexity. Specifically, the trade-off between complexity and performance is carefully examined, and it is found that by sacrificing a small amount of computational complexity, the scheme offers considerable gains in both energy and spectral efficiency.

(3) Simulations were conducted to compare the performance of the proposed scheme in the in-body channel environment within WBANs. The results show that the scheme demonstrates excellent bit error rate (BER) performance in the in-body channel environment. This indicates that the proposed scheme is well-suited for in-body environments. Additionally, the varying BER performance in the environments can be used to infer information about the current channel conditions, such as the specific type of channel environment and the position of the body. This provides a potential method for adaptive communication systems to dynamically adjust to environmental changes.

Section 2 introduced the channel model and presents the principle of the MIP-MDCSK scheme for impulsive noise in a channel. Section 3 computes EE, SE, and the complexity of MIP-MDCSK and compares them with corresponding schemes. Analysis of performances, simulation, and results are presented in Section 4, respectively. Section 5 concludes the paper.

## 2. Principle of the MIP-MDCSK Scheme

### 2.1. Channel Model

**Figure 1:**
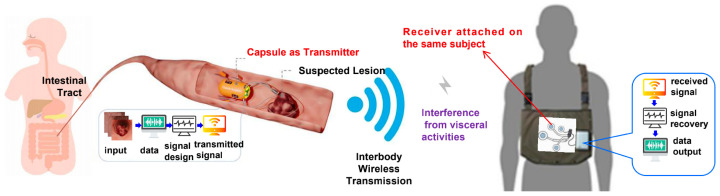
Endoscopic capsule implementation scheme.

The traditional endoscopic capsule communication transmission model is shown in Figure 1. In order to better address the issues of low cost and low power consumption, we have optimized the signal design scheme for the transmission module. For the pathway dissipation of the body’s surface and inner body, we can represent it using logarithmic functions. However, this is not applicable for intracellular signaling pathways. Nevertheless, the formula provided for pathway dissipation is essentially applicable to intracellular signaling pathways [35].

(1) $$P_{dB}\left(d\right)=P_{0_{dB}}+a{\left(\frac{d}{d_{0}}\right)}^{n},$$

where $d_{0}=5$ mm represents the reference distance, *d* denotes the distance from the skin surface to the chest cavity, with units in mm, and $P_{0}$ represents the pathway dissipation, measured in dB. *n* is the exponent of pathway dissipation, while *a* represents the fitted constant. Equation (1) provides a good fit for the dissipation of pathways within the body with a=11.6, n=0.5, and $p_{0}=6.3$ dB.

Based on the body surface profile of UWB and free space, the logarithmic distribution of narrowband channels is quite similar to the probability density function (PDF) of shadowing effects [36]. Computational results have demonstrated that the logarithmic distribution also accurately represents shadowing data for different distances in intracellular signaling pathways. Therefore, Equation (1) can be rewritten as

(2) $$P_{dB}\left(d\right)=P_{0_{dB}}+a{\left(\frac{d}{d_{0}}\right)}^{n}+N\left(\mu \left(d\right),\sigma \left(d\right)\right),$$

where *N* represents a random variable that follows a normal distribution, with a mean value of μ and a standard deviation of σ. The parameter *N* varies depending on the value of *d*.

In the channel model for in-body to on-body communication, Khalghi A proposed in reference [37] that the multipath gain and delay depth (*d*) follow linear Gaussian distributions with means $\mu_{l}\left(d\right)$ and $\mu_{l}^{\prime }\left(d\right)$ for the *l*-th multipath, and variances $\sigma_{l}\left(d\right)$ and $\sigma_{l}^{\prime }\left(d\right)$, respectively. The Moreover, $\mu_{l}\left(d\right)$ can be expressed as

(3) $$\begin{array}{c} \mu_{l}\left(d\right)=\omega_{0}\left(d\right)e^{-(l-1)\lambda (d)}, \end{array}$$

where the $\omega_{0}\left(d\right)$ is the peak average power, and λ(d) is the exponential decay factor of peak power. For a given depth d, $\mu_{l}^{\prime }\left(d\right)$ is always a constant. Similarly, $\sigma_{l}^{\prime }\left(d\right)$ can be expressed as

(4) $$\begin{array}{c} \sigma_{l}^{\prime }\left(d\right)=\Sigma_{0}\left(d\right)e^{-(l-1)\Lambda (d)}, \end{array}$$

where the $\Sigma_{0}\left(d\right)$ is the Peak average power, Λ(d) is the exponential decay factor of peak power.

### 2.2. IP-MDCSK and General MIP-MDCSK Sharing One Reference Signal over In-Body Channel

#### 2.2.1. IP-MDCSK

The system based on IP-MDCSK with $n_{c}$ bits is achieved by index modulation, and the $m_{c}$ bits are achieved by MDCSK. Thus, the total number of transmitted bits is $m_{c}+n_{c}$, and the symbol duration is $(1+2^{n_{c}})R$, with $\log_{2}M=m_{c}$. The chaotic generator generates the reference signal $c_{x,k}$, with $c_{y,k}$ being its Hilbert transform. Thus, the transmitted signal is $t_{s}=a_{s}c_{x,k}+b_{s}c_{y,k}$, where s=1,…M is the symbol in the signal space [27], and $a_{s}$ and $b_{s}$ are the orthogonal coordinates of the chaotic symbol $t_{s}$. Assume that $a_{l}$ time slots are chosen from a total of *P* time slots to transmit the information-bearing signals, while the remaining $P-a_{l}$ time slots remain unused. $a_{l}$ takes values within the range from 1 to $a_{l}$, where $a_{l}$ is less than P. As a result, the total is given by $u=\sum_{p=1}^{P}\frac{P!}{a_{l}!\left(P-a_{l}\right)!}$ for indexes, and t is the number of indexes selected. Hence, the output of the transmitter is expressed as

(5) $$\begin{array}{cc} \; & s_{\mathrm{xo}}=\left[\underset{reference}{\underset{︸}{c_{x}}},\underset{information-bearing}{\underset{︸}{i_{x}}}\right], \end{array}$$

where $i_{x}=s_{IP}⨂(a_{s}c_{x}+b_{s}c_{y})$, which is used to carry the reference signal. $\sum_{k=1}^{R}c_{x,k}^{2}=\sum_{k=1}^{R}c_{y,k}^{2}=1$, $\sum_{k=1}^{R}c_{x,k}c_{y,k}=0$, $a_{s}^{2}+b_{s}^{2}=1$. $c_{x}=\left[c_{x,1},c_{x,1},\ldots ,c_{x,R}\right]$ denotes an R-length chaotic signal sequence, and 2R=β is the spreading factor. The overall number of possible transmitted information-bearing signals is $U=2^{a_{l}}\sum_{a_{l}=1}^{P}\frac{P!}{a_{l}!\left(P-a_{l}\right)!}$. Assuming $n_{c}=2,t=2$, the matrix for index position is expressed as

(6) $$\begin{array}{cc} \; & M=\left[\begin{array}{cccc} +1 & 0 & 0 & 0\\ 0 & 0 & 0 & +1\\ -1 & 0 & 0 & 0\\ 0 & 0 & 0 & -1\\ 0 & 0 & +1 & 0\\ 0 & +1 & 0 & 0\\ 0 & -1 & 0 & 0\\ 0 & 0 & -1 & 0 \end{array}\right], \end{array}$$

where $s_{IP}$ is the active row selected from the *M* matrix, represented as $s_{IP}=M_{al}$, $a_{l}$ is the index position selected from the matrix *M*. The ⨂ is the Kronecker product.

Assuming the modulated symbols are transmitted over a PLC channel with multipath, and the *x*-th received signal is obtained as

(7) $$r_{x,k}=\sum_{l=1}^{L}\alpha_{l}\delta \left(k-\tau_{l}\right)⨂s_{x,k}+W_{k},$$

where δ(.) is the impulse function, $\alpha_{l}$ and $\tau_{l}$ are the gain and delay of the lth path, respectively, and *L* is the number of paths. $W_{k}$ is the noise for in-body whose PDF follows Equation (2). When $\alpha_{l}$ and *L* paths are one, the channel has only one path.

At the receiver, both MDCSK and index positions are detected. For the index part, position information-bearing signals $r_{inf}=r_{al}$. After obtaining the position of information-bearing signals, the reference signal ${\tilde{c}}_{x,k}$ on the receiver correlates with the received information-bearing signal ${\tilde{t}}_{s}$, and then the $z_{a,o}$ is obtained. Moreover, $z_{b,o}$ can be obtained by correlating ${\tilde{t}}_{s}$ and ${\tilde{c}}_{y}$, where ${\tilde{c}}_{y,k}$ is the Hilbert transform of ${\tilde{c}}_{x,k}$. The received symbol is decided by $\arctan(z_{a,o}/z_{b,o})$. We assume that the delay τ is much shorter than the chaotic sequence duration, i.e., 0<τ<<R. Thus, the inter-symbol interference (ISI) present can be neglected [27]. After finding the indexed position, the decision variable of $z_{a,o}$ and $z_{b,o}$ can be written as

(8) $$\begin{array}{c} z_{a,o}=\sum_{k=1}^{R}\left(\sum_{l=1}^{L}\alpha_{l}c_{x,k}+W_{k}\right)(\sum_{l=1}^{L}\alpha_{l}\left(a_{s}c_{x,k}+b_{s}c_{y,k}\right)\\ +W_{k+R}), \end{array}$$

(9) $$\begin{array}{c} z_{b,o}=\sum_{k=1}^{R}\left(\sum_{l=1}^{L}\alpha_{l}c_{y,k}+\tilde{W_{k}}\right)(\sum_{l=1}^{L}\alpha_{l}\left(a_{s}c_{x,k}+b_{s}c_{y,k}\right)\\ +W_{k+R}). \end{array}$$

Similarly, when $a,b\neq a_{l}$, the corresponding decision variable can be expressed as

(10) $$\begin{array}{c} z_{ao,n}=\sum_{k=1}^{R}\left(\sum_{l=1}^{L}\alpha_{l}c_{x,k}+W_{k}\right)W_{k+R}, \end{array}$$

(11) $$\begin{array}{c} z_{bo,n}=\sum_{k=1}^{R}\left(\sum_{l=1}^{L}\alpha_{l}c_{y,k}+\tilde{W_{k}}\right)W_{k+R}, \end{array}$$

$a_{l}$ can be detected using the maximum comparator as

(12) $$\begin{array}{cc} \; & {\hat{a}}_{l}=\arg\max_{m=1,\ldots P}\left(\right|z_{a,o}+z_{a,n}\left|\right), \end{array}$$

where **$W_{k}$** is caused by the reference signal and **$\tilde{W_{k}}$** is caused by the Hilbert transform of **$W_{k}$**.

#### 2.2.2. A General MIP-MDCSK with Shared Reference Signal

**Figure 2:**
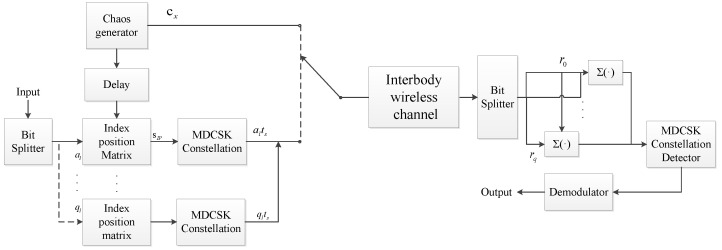
Block diagram of the MIP-MDCSK scheme for data transmission module of Endoscopic Capsule.

The IP-MDCSK can improve the BER performance at the expense of bandwidth efficiency [38]. Considering the bandwidth utilization, MIP-MDCSK is proposed. The block of the scheme is shown in Figure 2, which has *t* information-bearing signals mapped to *t* different indexes that are sharing the same reference signal. The total transmitted bits are $m_{c}+N_{c}$, where $N_{c}=tn_{c}$. Similarly, the output of the transmitter for MIP-MDCSK is

(13) $$\begin{array}{cc} \; & s_{x}=\left[\underset{reference}{\underset{︸}{c_{x}}},\underset{information-bearing}{\underset{︸}{{\mathrm{mi}}_{x}}}\right], \end{array}$$

where ${\mathrm{mi}}_{x}=s_{MIP}⨂\left(a_{s}c_{x}+b_{s}c_{y}\right)$, and $s_{MIP}$ consists of *t* independent $s_{IP}$ components, given as $s_{MIP}=M_{a_{l}},M_{b_{l}},\ldots ,M_{q_{l}}$, the $b_{l}$ and $q_{l}$ are also the index positions selected from the matrix *M*.

Where the $b_{l},\ldots$ is also the position index modulation symbol. The total number of possible transmitted information-bearing signals is $UU=\sum_{\omega_{l}}^{\Omega_{l}}2^{\omega_{l}}\sum_{\omega_{l}=1}^{P}\frac{P!}{\omega_{l}!\left(P-\omega_{l}\right)!}$.

At the receiver, the detector detects the positions of multi-index with maximum comparator, where the $a_{l}$ and $b_{l}$ are obtained. Then, the remaining $m_{c}$ bits of information are obtained by demodulation of the MDCSK.

## 3. Performance Analysis

### 3.1. Energy Efficiency (EE), and Spectral Efficiency (SE)

We assume that all systems utilize the same chaotic signal with an *R*-length and unit energy as a reference. In the proposed scheme, the symbol energy $E_{k}$ is expressed as

(14) $$\begin{array}{c} E_{k}=E_{data1}+E_{data2}+\ldots +E_{ref}, \end{array}$$

where $E_{ref}$ is the energy of the reference sequence. Thus, $E_{data1}=E_{data2}=\ldots =E_{ref}$ and $E_{k}=\left(q+1\right)E_{ref}$. It is obvious that $E_{ref}$ is used to transmit the reference signal while $E_{k}$ is used to transmit $m_{c}+tn_{c}$ bits. Thus the energy required for transmitting one bit, which is compared with reference part $E_{ref}$, is:

(15) $$\begin{array}{c} E_{b}=\frac{E_{k}}{m_{c}+qn_{c}}, \end{array}$$

Subsequently, the energy efficiency (EE), i.e., the number of bits per unit of data energy, can be written as

(16) $$\begin{array}{c} EE=\frac{E_{data1}+E_{data2}+\ldots E_{datat}}{E_{b}}=\frac{t(m_{c}+qn_{c})}{\left(q+1\right)}. \end{array}$$

Table 1 presents the energy efficiency (EE) of various chaotic modulation schemes. As observed in the table, DCSK transmits only a single bit across two time slots, resulting in an EE of 1/2. In contrast, the traditional MDCSK transmits $m_{c}$ bits within the same time period. The table also highlights that 2IP-MDCSK achieves a significantly higher EE compared to IP-MDCSK, MCS-DCSK, and MDCSK. Furthermore, the EE values for these schemes improve as the value of M increases.

A comparison of spectral efficiency (SE) between the proposed MIP-MDCSK and other relevant schemes is provided in Table 2. Let B represent the bandwidth. The SE for MIP-MDCSK is given by equation $SE_{MIP-MDCSK}=\frac{m_{c}+tn_{c}}{R(1+q2^{n_{c}+1})B}$ [17]. Similar expressions for the SE of the other schemes are also included in the table. The results indicate that the proposed MIP-MDCSK consistently offers a higher SE than IP-MDCSK, and it outperforms MCS-DCSK when the parameters are appropriately chosen. However, it falls short of traditional MDCSK in terms of SE. In conclusion, the proposed system achieves a balanced trade-off between SE, EE, and BER performance.

### 3.2. Complexity Analysis Comparison with MDCSK

This section presents a comparison of the hardware computational complexity of MIP-MDCSK with that of the corresponding MDCSK schemes, all of which operate at the same modulation order (M). The details of the components involved in the transmission and reception processes for MIP-MDCSK, MCS-DCSK, and MDCSK are summarized in Table 3 and Table 4, respectively. To achieve M-ary modulation, all schemes require the use of filtration twice, both at the transmitter and receiver, in order to generate orthogonal signals. Additionally, the complexity of MIP-MDCSK and MCS-DCSK systems is increased due to the implementation of position and Walsh code selection techniques, making them more intricate than the MDCSK scheme. Furthermore, the delay units in the transmitter and multipliers in the receiver of MIP-MDCSK are more sophisticated compared to those in the other schemes. As a result, the increased computational complexity in MIP-MDCSK is a trade-off for its enhanced BER performance.

## 4. Numerical Result

This section presents an analysis of the performance of systems utilizing the proposed constellation scheme under various system parameters. The distribution parameters corresponding to different distances are shown as Table 5.

**Figure 3:**
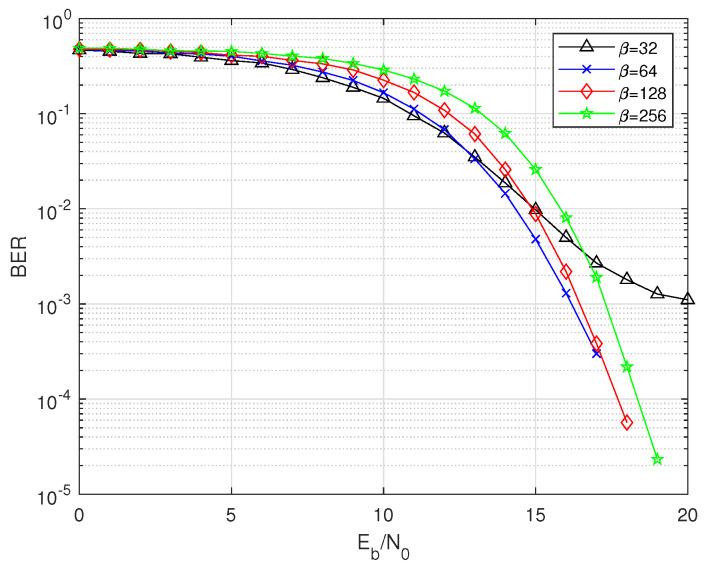
BER comparison of 2IP-MDCSK for β over in-body channel.

To investigate the BER performance of the system in an in-body channel environment, we examine the effects of parameters such as β, *M*, *n*, and the in-body channel parameter depth. Figure 3 demonstrates the performance of the 2IP-MDCSK system under in-body channel conditions with system parameters set as depth = 80 mm, *M* = 4, and *n* = 2. From the figure, it is evident that the overall BER performance deteriorates as β increases. However, when β = 32, the BER performance is significantly worse compared to other values. This can be attributed to the characteristics of the chaotic signal. At smaller β values, the system struggles to effectively exhibit the correlation properties, leading to a higher likelihood of bit errors.

**Figure 4:**
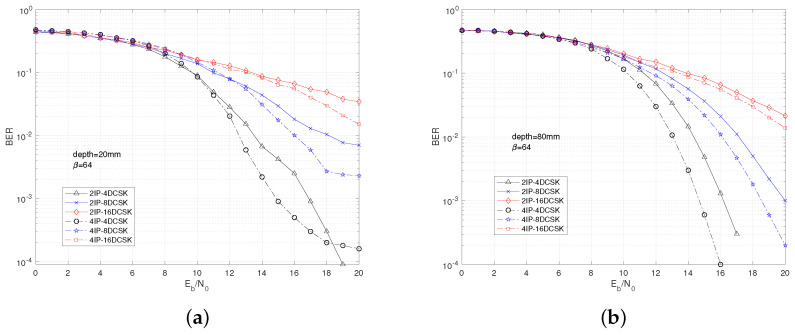
BER comparison of MIP-MDCSK for in-body channel. (**a**) depth = 20 mm. (**b**) depth = 80 mm.

Figure 4 illustrates the impact of different values of M and $n_{c}$ on the performance of the MIP-MDCSK system under varying depth conditions within an in-body environment. Overall, as M increases, the BER performance deteriorates, while an increase in $n_{c}$ leads to improved system performance. Moreover, the influence of M on the BER performance is significantly more pronounced than that of $n_{c}$. For instance, in Figure 4a, at a BER of $10^{-2}$, the gap between 2IP-4DCSK and 4IP-4DCSK is 1 dB, whereas the gap between 2IP-4DCSK and 2IP-8DCSK at the same BER is 2.5 dB. This clearly demonstrates that the constellation parameters have a much greater impact on the BER performance than the IP components. Regarding the effect of depth on the system, a comparison from Figure 4a,b shows that when depth = 80 mm, the overall BER performance is better than when depth = 20 mm. At a BER of $10^{-4}$, the 2IP-4DCSK system shows a gain of approximately 1 dB at depth = 80 mm compared to depth = 20 mm. This suggests that a greater depth brings the system closer to the in-body channel environment, indicating that the system is better suited for in-body channel conditions.

**Figure 5:**
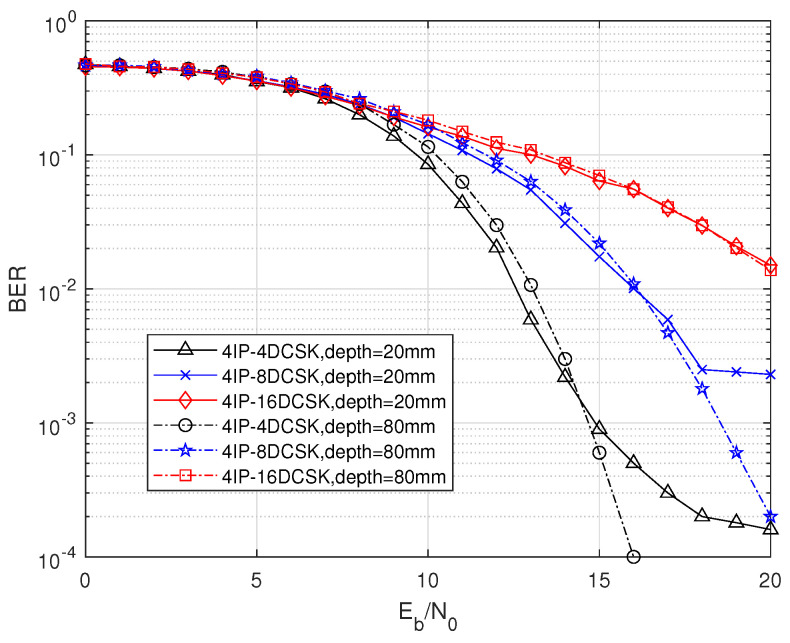
BER comparison of MIP-MDCSK for depth in in-body channel, with β=64.

Further discussion on the impact of depth on the system is provided in Figure 5. As shown in the figure, the BER performance at depth = 80 mm is significantly better than at depth = 20 mm. At depth = 20 mm, when the $E_{b}/N_{0}$ increases to a certain level, the system gradually experiences an error floor. The SNR value at which the error floor begins to appear shifts to a lower value as M decreases. However, for the MIP-MDCSK system itself, its performance is optimal when M = 4, and the BER performance deteriorates as M increases, which is contrary to the behavior observed with the error floor. In contrast, at depth = 80 mm, the system does not exhibit a noticeable error floor, further supporting the suitability of this system model for channels that closely resemble in-body environments.

**Figure 6:**
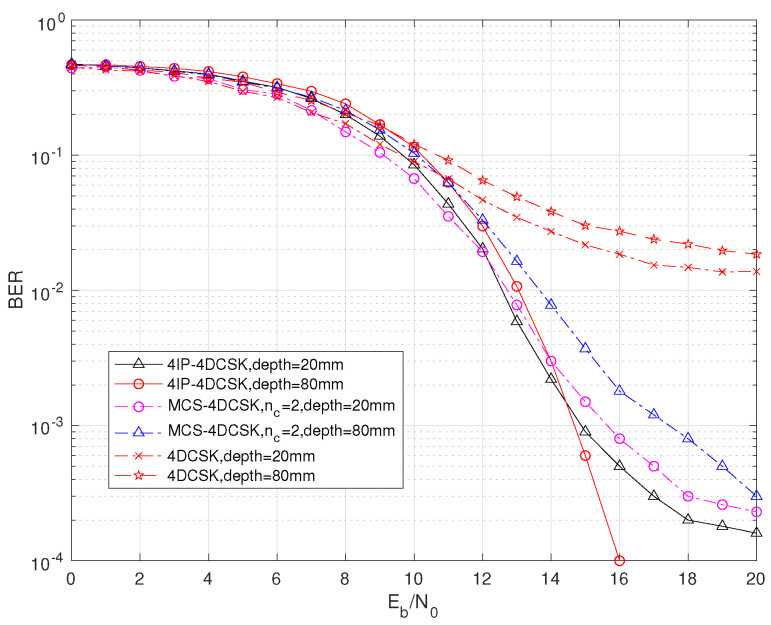
BER comparison of 2IP-MDCSK among MDCSK and MCS-MDCSK, with β = 64 over in-body channel.

To further discuss the proposed solution, it is compared with MCS-MDCSK and MDCSK, as shown in Figure 6. According to the graph, the proposed scheme has the best bit error rate performance under different depth parameters. From the other two schemes, it is known that at depth = 80 mm, their bit error rate performance is better at depth = 20 mm than when the $E_{b}/N_{0}$ is small and worse than at depth = 20 mm when the $E_{b}/N_{0}$ is large. The intersection points of the proposed MIP-MDCSK appear earlier.

## 5. Conclusions

This paper addresses issues such as high power consumption, significant signal interference, and low data transmission rates in capsule endoscopy communication. It proposes a modulation optimization scheme (MIP-MDCSK) aimed at improving performance through three main aspects: BER performance, EE, and SE. The performance of the proposed scheme is evaluated for WBANs.

Through Monte Carlo simulations, the BER is evaluated in the in-body channel. The results reveal that the in-body channel yields favorable BER performance, especially as the $n_{c}$ value increases, indicating better robustness against noise. The gap between 2IP-4DCSK and 4IP-4DCSK is 1 dB, whereas the gap between 2IP-4DCSK and 2IP-8DCSK at the same BER is 2.5 dB at a BER of $10^{-2}$. Moreover, the BER of the system can be significantly reduced to $10^{-5}$, indicating that it meets the BER performance requirements of in-body channel devices that need to be synchronized in medical image transmission environments.

Therefore, this optimized solution can address the low-cost and low-power communication transmission of endoscopic capsules while improving transmission performance, thus promoting the widespread adoption and development of endoscopic capsules. 

## Figures and Tables

**Table 1:** Comparisons of energy efficiency (EE).

	EE			
Modulation	MIP-MDCSK	IP-MDCSK	MCS-DCSK	MDCSK
expression				
	$\frac{q(m_{c}+tn_{c})}{t+1}$	$\frac{m_{c}+n_{c}}{2}$	$\frac{n_{c}+1}{2}$	$\frac{m_{c}}{2}$
numerical value				
set $m_{c}=2,n_{c}=2,t=2$	8	4	1.5	1
set $m_{c}=3,n_{c}=3,t=3$	12	3	2	1.5

**Table 2:** Comparisons of spectral efficiency (SE).

	SE			
Modulation	MIP-MDCSK	IP-MDCSK	MCS-DCSK	MDCSK
expression				
	$\frac{m_{c}+tn_{c}}{R(1+2^{n_{c}+1})B}$	$\frac{m_{c}+n_{c}}{R(1+2^{n_{c}})B}$	$\frac{n_{c}}{(2^{n_{c}}+1)RB}$	$\frac{m_{c}}{2RB}$
numerical value				
$m_{c}=2,n_{c}=2,t=2,R=B=1$	0.89	0.8	0.4	1
$m_{c}=3,n_{c}=3,t=3,R=B=1$	0.71	0.67	0.33	1.5

**Table 3:** Comparisons of Transmitter Computational Complexity.

Modulation	Adder	Multipliers	Delay Units	Modulator	Selection
MIP-MDCSK	0	*M*	*M*	comparator, Hilbert filter	position
MDCSK	0	0	1	Hilbert filter	none
MCS-DCSK	$N_{b}+1$	$N_{b}+1$	0	code shift detector, Hilbert filter	Walsh code

**Table 4:** Comparisons of Receiver Computational Complexity.

Modulation	Multipliers	Detector	Selection
MIP-MDCSK	2	maximum energy comparator, Hilbert filter	position
MDCSK	2	Hilbert filter	none
MCS-DCSK	$N_{b}+1$	code shift detector, Hilbert filter	Walsh code

**Table 5:** The distribution parameters corresponding to depth [39].

Depth (mm)	(μ, σ)
120	(2.7, 4.9)
80	(8.2, 6.6)
40	(5.3, 8.1)
20	(3.5, 6.2)

## Data Availability

See Liu, W. Performance Analysis of Differential Chaotic Shift Keying Ultra-Wideband Systems in In-Body Channels. Master’s Thesis, Xiamen University, Xiamen, China, 2022.
